# Fumarates modulate microglia activation through a novel HCAR2 signaling pathway and rescue synaptic dysregulation in inflamed CNS

**DOI:** 10.1007/s00401-015-1422-3

**Published:** 2015-04-29

**Authors:** Benedetta Parodi, Silvia Rossi, Sara Morando, Christian Cordano, Alberto Bragoni, Caterina Motta, Cesare Usai, Brian T. Wipke, Robert H. Scannevin, Giovanni L. Mancardi, Diego Centonze, Nicole Kerlero de Rosbo, Antonio Uccelli

**Affiliations:** Department of Neurology, Rehabilitation, Ophthalmology, Genetics, Maternal and Child Health, University of Genoa, Genoa, Italy; Department of Neurosciences, Tor Vergata University of Rome, Rome, Italy; National Research Council, Institute of Biophysics, University of Genoa, Genoa, Italy; Biogen, Cambridge, MA USA; Centre of Excellence for Biomedical Research, University of Genoa, Genoa, Italy

**Keywords:** Experimental autoimmune encephalomyelitis, Multiple sclerosis, Microglia, Hydroxycarboxylic acid receptor 2, Synaptopathy, Neuroinflammation, Neuroprotection

## Abstract

Dimethyl fumarate (DMF), recently approved as an oral immunomodulatory treatment for relapsing-remitting multiple sclerosis (MS), metabolizes to monomethyl fumarate (MMF) which crosses the blood–brain barrier and has demonstrated neuroprotective effects in experimental studies. We postulated that MMF exerts neuroprotective effects through modulation of microglia activation, a critical component of the neuroinflammatory cascade that occurs in neurodegenerative diseases such as MS. To ascertain our hypothesis and define the mechanistic pathways involved in the modulating effect of fumarates, we used real-time PCR and biochemical assays to assess changes in the molecular and functional phenotype of microglia, quantitative Western blotting to monitor activation of postulated pathway components, and ex vivo whole-cell patch clamp recording of excitatory post-synaptic currents in corticostriatal slices from mice with experimental autoimmune encephalomyelitis (EAE), a model for MS, to study synaptic transmission. We show that exposure to MMF switches the molecular and functional phenotype of activated microglia from classically activated, pro-inflammatory type to alternatively activated, neuroprotective one, through activation of the hydroxycarboxylic acid receptor 2 (HCAR2). We validate a downstream pathway mediated through the AMPK–Sirt1 axis resulting in deacetylation, and thereby inhibition, of NF-κB and, consequently, of secretion of pro-inflammatory molecules. We demonstrate through ex vivo monitoring of spontaneous glutamate-mediated excitatory post-synaptic currents of single neurons in corticostriatal slices from EAE mice that the neuroprotective effect of DMF was exerted on neurons at pre-synaptic terminals by modulating glutamate release. By exposing control slices to untreated and MMF-treated activated microglia, we confirm the modulating effect of MMF on microglia function and, thereby, its indirect neuroprotective effect at post-synaptic level. These findings, whereby DMF-induced activation of a new HCAR2-dependent pathway on microglia leads to the modulation of neuroinflammation and restores synaptic alterations occurring in EAE, represent a possible novel mechanism of action for DMF in MS.

**Electronic supplementary material:**

The online version of this article (doi:10.1007/s00401-015-1422-3) contains supplementary material, which is available to authorized users.

## Introduction

Dimethyl fumarate (DMF) was recently approved as a first-line oral therapy for multiple sclerosis (MS), an inflammatory autoimmune disease of the CNS characterized by demyelination and axonal loss, following phase III clinical trials which showed that DMF successfully reduced relapse rate and the number of new lesions on MRI [13, 18], and increased time to progression of disability in relapsing-remitting MS patients [18]. The mode of action of DMF is only partially understood, but in vitro and in vivo studies suggest that DMF and its active metabolite, monomethyl fumarate (MMF), modulate immune cells. In particular, DMF was shown to switch T cells from a Th1- to a Th2-type profile in vitro and in vivo [27], an effect possibly related to the induction of type II dendritic cells that promote Th2-type T cells [16], and/or inhibition of dendritic cell maturation, thereby affecting Th1 and Th17 cell differentiation [34]. Studies in experimental autoimmune encephalomyelitis (EAE), an experimental model of MS, have demonstrated that oral administration of DMF in EAE mice ameliorated the clinical course of the disease, both when applied as a preventive (prior to disease onset) or a therapeutic (at disease chronic phase) treatment [28, 45]. Upon oral administration, DMF is rapidly metabolized to MMF, which crosses the blood–brain barrier and achieves detectable levels in the CNS [19, 28], supporting the potential for a direct effect on CNS resident cells. Several studies have postulated that fumarates, as thiol-reactive electrophilic agents, could act through the nuclear factor (erythroid-derived 2)-like 2 (Nrf2) pathway, which mediates induction of phase II genes by thiol-reactive electrophiles, thereby playing a major role in cell and tissue defense against oxidative stress [54]. In vitro analysis of the effect of MMF on neurons and astrocytes exposed to oxidative stress showed that MMF induced the activation of the Nrf2 oxidative response pathway in these cells [43], an effect validated by ex vivo immunohistochemical analysis of spinal cord tissue from DMF-treated EAE mice showing increased Nrf2 protein expression in NeuN-positive cells [28]. However, Nrf2-independent mechanisms might also be involved in the immunomodulatory effect of DMF [46] and a recent study showed that the hydroxycarboxylic acid receptor 2 (HCAR2), a G protein-coupled membrane receptor, was required for the therapeutic effects of DMF in EAE, possibly through inhibition of neutrophil infiltration [10]. DMF has been shown to also affect astrocytes and microglia, inhibiting their production of neuroinflammatory mediators [55]. In chronic inflammatory diseases such as MS, microglia become activated and release pro-inflammatory cytokines and stress-associated molecules leading to neurodegeneration and alteration of synaptic transmission [9]. However, modulation of microglia activation towards an alternatively activated phenotype, favoring neuroprotection, can modify the outcome of some experimental models of neurological diseases [17]. Here, we show that in vitro application of MMF to microglia switches the phenotype of activated microglia from pro-inflammatory to anti-inflammatory, with downregulation of pro-inflammatory markers and upregulation of neuroprotective ones. We demonstrate that microglia express HCAR2 and that MMF binding to HCAR2 triggers a pathway driven by the AMPK/Sirt axis resulting in inhibition of NF-κB and of subsequent pro-inflammatory cytokine production. Finally, we validate that DMF administration to mice with EAE results in upregulation of microglia markers associated with neuroprotection and mitigates deleterious alterations in synaptic transmission.

## Materials and methods

### Microglia culture and treatments

The murine microglial cell line N9 developed by Prof. P. Ricciardi-Castagnoli and kindly provided by Prof. E. Zocchi, Genoa, Italy, was obtained by immortalization of murine microglial cells from embryonic day 13 with the 3RV retrovirus carrying an activated vmyc oncogene [39]. N9 cells were grown in Iscove’s Modified Dulbecco’s Medium (IMDM, Lonza) supplemented with 5 % fetal bovine serum, 1 % penicillin and streptomycin, and 50 nM β-mercaptoethanol, and cultured in a humidified 5 % CO_2_ atmosphere at 37 °C. N9 cells seeded in 6-well plates (8 × 10^5^ cells/well) in 3 ml IMDM were activated with 1 μg/ml LPS (Sigma-Aldrich) for various times, as indicated in the text and/or figure legends and treated concomitantly with MMF (1 μM in 0.001 % dimethyl sulfoxide final concentration, except where indicated). Where relevant, N9 cells were incubated with anti-HCAR2 antibody (anti-mouse HCAR2, Novus Biologicals, NBP1-00875; 1 μg/ml) for 1 h prior to activation with LPS in the presence or absence of MMF. Treatments with EX527 and resveratrol (Resv; Sigma-Aldrich) are detailed in the legend to the relevant figure.

### Cytotoxicity assay

N9 cells were seeded in 96-well plates (5 × 10^4^ in 200 μl of IMDM) and treated, or not, with different concentrations of MMF (1 μM, 10 μM, 100 μM) for 24 h. The medium was then replaced by serum-free IMDM containing 10 % MTT (3-(4,5-dimethylthiazol-2-yl)-2,5-diphenyltetrazolium Bromide, Sigma-Aldrich) and cell viability was measured by spectrophotometric absorbance at 570 nm. Percentage viability was calculated as optical density (OD) of treated sample/OD of untreated sample × 100.

### RNA isolation and real-time PCR

Total RNA was isolated from N9 cells and EAE brains using Trizol reagent (Life Technologies) according to the manufacturer’s instructions. cDNA was synthetized from 1 μg RNA using the Transcriptor First Strand cDNA Synthesis Kit (Roche). Real-time PCR was performed using a LightCycler 480 (Roche) in duplicates with a final reaction volume of 20 μl containing 50 ng cDNA, 1 μl of each primer pair (20 μM), and 10 μl of LightCycler 480 SYBR Green I Master Mix (Roche). Amplification of glyceraldehyde 3-phosphate dehydrogenase (Gapdh) was used to normalize the expression data. The primer pairs for the indicated genes were synthetized by Tib Molbiol according to the following sequences: Tnf, forward primer: 5′-TCTTCTCATTCCTGCTTGTGG-3′, reverse primer: 5′-GGTCTGGGCCATAGAACTGA-3′; Il1b, forward primer: 5′-AGTTGACGGACCCCAAAAG-3′, reverse primer: 5′-TTTGAAGCTGGATGCTCTCAT-3′; Spi1, forward primer: 5′-GGGATCTGACCAACCTGGA-3′, reverse primer: 5′-AACCAAGTCATCCGATGGAG-3′; Nos2, forward primer: 5′-TGAACTTGAGCGAGGAGCA-3′, reverse primer: 5′-TTCATGATAACGTTTCTGGCTCT-3′; Hmox1, forward primer: 5′-GTCAAGCACAGGGTGACAGA-3′, reverse primer: 5′-ATCCCTGCAGCTCCTCAAA-3′; Cx3cr1, forward primer: 5′-AAGTTCCCTTCCCATCTGCT-3′, reverse primer: 5′-CAAAATTCTCTAGATCCAGTTCAGG-3′, Cd200r, forward primer: 5′-AAATGCAAATTGCCAAAATTAGA-3′, reverse primer: 5′-GTATAGTAGCATAAGGCTGCATTT-3′; Nr4a2, forward primer: 5′-TCAGAGCCCACGTCGATT-3′, reverse primer: 5′-TAGTCAGGGTTTGCCTGGAA-3′; Igf1, forward primer: 5′-AGCAGCCTTCCAACTCAATTAT-3′, reverse primer: 5′-GAAGACGACATGATGTGTATCTTTATC-3′, Arg1, forward primer: 5′-GAATCTGCATGGGCAACC-3′, reverse primer: 5′-GAATCCTGGTACATCTGGGAAC-3′, Retnla1, forward primer: 5′-CCCTCCACTGTAACGAAGACTC-3′, reverse primer: 5′-CACACCCAGTAGCAGTCATCC-3′; Trem2, forward primer: 5′-TGGGACCTCTCCACCAGTT-3′, reverse primer: 5′-GTGGTGTTGAGGGCTTGG-3′; Lgals3, forward primer 5′-AAGGAGAACAGGGCAAAGG-3′, reverse primer: 5′- TGGACTTGCAGGGCTTCT-3′; Mrc1, forward primer: 5′-CCACAGCATTGAGGAGTTTG-3′, reverse primer: 5′-ACAGCTCATCATTTGGCTCA-3′; Gapdh: forward primer: 5′-AATCTCCACTTTGCCACTGC-3′, reverse primer: 5′-ATGGTGAAGGTCGGTGTGA-3′.

### Phagocytosis assay

N9 cells (8 × 10^5^) plated on 35-mm glass-bottom cell culture dishes were activated or not with LPS in the presence or absence of MMF. After 24 h, cells were incubated with 10 μl of Fluoresbrite microspheres (Polysciences Inc.) for 30 min at 37 °C. The phagocytic activity was blocked by addition of ice-cold PBS and the cells were washed twice with ice-cold PBS. Several hundred N9 cells were analyzed by confocal microscopy (TCS SP5, LEICA Microsystems) and the extent of phagocytosis was quantified as follows: for every cell analyzed, the area of the maximal confocal section was measured, the fluorescence emitted by the beads was binarized in the same section and the total area of the fluorescent surface was measured; the ratio between the fluorescent area and the cell section area was calculated and phagocytosis was quantified as the area occupied by the microspheres.

### Fluorimetric determination of intracellular Ca^2+^ concentration ([Ca^2+^]_i_)

N9 cells (5 × 10^5^) were plated on 20-mm-diameter coverslips and activated, or not, with LPS in the presence or absence of MMF for 24 h. Cells were then incubated with the fluorescent calcium indicator Fura-2-acetoxymethyl ester (Fura 2 AM) for 45 min at 37 °C, and [Ca^2+^]_i_ was measured as fluorescent intensity on an inverted microscope (Zeiss IM35, Zeiss) as described elsewhere [63].

### Immunochemistry and confocal microscopy

N9 cells were seeded on 20-mm glass coverslip (5 × 10^5^ cells per coverslip) overnight. The next day, cells were fixed with 4 % paraformaldehyde (PFA) for 10 min at room temperature and permeabilized with 0.25 % Triton X 100 for 10 min. After blocking in 1 % bovine serum albumin (BSA) for 15 min at room temperature, cells were stained for 1 h in humid chamber with primary antibodies: rabbit anti-HCAR2 (1:200, Novus Biologicals) and rat anti-CD11b (1:100, Abnova, ab8879) in 1 % BSA. Cells were then incubated with Alexa 488-labeled anti-rabbit and Alexa 594-labeled anti-rat secondary antibodies (both used at 1:500, Life Technologies, A-11034 and A-11007) at room temperature for 30 min and the nuclei were identified with 4,6-diamidino-2-phenylindole (DAPI). Images were analyzed by confocal microscopy (TCS SP5, LEICA Microsystems GmbH).

### Western blotting

N9 cells were lysed in RIPA buffer containing protease inhibitor cocktail (Roche) and phosphatase inhibitor cocktail (Sigma-Aldrich). Protein samples (30 μg) were electrophoresed on a 4–12 % gradient polyacrylamide pre-cast gel (Life Technologies), using Bolt^®^ Mini Gel Tank (Life Technologies) and transferred to nitrocellulose membrane (BioRad) using XCell II™ Blot Module (Life Technologies). Membranes blocked with 5 % non-fat milk in PBS/0.1 % Tween 20 were incubated with primary antibodies overnight, washed and incubated with horseradish peroxidase-conjugated anti-rabbit IgG (1:4000, Merck-Millipore, AP307P) for 1 h, and visualized using ECL Plus (Thermo Fisher Scientific). Quantification of relative protein amounts was performed by densitometric analysis using ImageJ software (NIH), normalized to a loading control protein, growth factor receptor-bound protein 2 (GRB2). Primary rabbit antibodies used were: anti-phospho-5′ adenosine monophosphate-activated protein kinase (AMPK) Thr172 (1:1000, Cell Signaling, 2535), anti-AMPK (1:3000, Cell Signaling, 2603), anti-acetyl-nuclear factor kappa-light-chain-enhancer of activated B cells (NF-κB) Lys310 (1:1000, Cell Signaling, 3045), anti-NF-κB (1:3000, Cell Signaling, 4764), and anti-GRB2 (1:2000, Abcam, ab32111) antibodies.

### Nicotinamide adenine dinucleotide (NAD)+ quantification

N9 cells seeded overnight in 96-well plates (5 × 10^4^ cells/well) were incubated, or not, with anti-HCAR2 antibody (aHCAR2) for 1 h and activated with LPS, in the presence or absence of MMF for 30 min. The concentration of intracellular NAD+/NADH was determined in the lysed cells by a colorimetric reaction using NAD+/NADH Cell-Based Assay Kit (Cayman Chemical).

### EAE induction and DMF treatment

Female C57BL/6J mice, 6–8 weeks old, weighing 18.5 ± 1.5 g, were purchased from Harlan Italy. All animals were housed in pathogen-free conditions and treated according to the Italian and European guidelines (Decreto Legislativo 4 marzo 2014, n. 26, legislative transposition of Directive 2010/63/EU of the European Parliament and of the Council of 22 September 2010 on the protection of animals used for scientific purposes), with food and water ad libitum. The research protocol was approved by the Ethical Committees for Animal Experimentation of the University of Genoa and Tor Vergata University of Rome. After a 1-week period of adaptation to the animal house, mice were immunized as described before [59] by subcutaneous injection at two sites in the flank with an emulsion of 200 μg myelin oligodendrocyte glycoprotein (MOG) peptide 35–55 (Espikem) in incomplete Freund adjuvant (IFA; Difco) containing 300 μg Mycobacterium tuberculosis (strain H37Ra; Difco). Mice were injected in the tail vein with 400 ng pertussis toxin (Sigma-Aldrich) immediately and 48 h after immunization. The mice were scored daily for clinical manifestations of EAE on a scale of 0–5 [59].

The DMF suspension (15 mg/ml) in 0.8 % hydroxypropyl methylcellulose was prepared weekly and kept at 4 °C under constant stirring. DMF (200 μl corresponding to a dose of 150 mg/kg body weight) was administered daily by oral gavage through a bulb-tipped curved gastric gavage needle by trained operators from the onset of clinical symptoms until day 30. Control EAE animals were gavaged with vehicle alone. Mice were treated and daily assessed in a random order. For sampling and at completion of the experiment, mice were euthanized by gradual-fill CO_2_ exposure.

### Electrophysiological studies

Electrophysiological experiments were performed as previously described [9, 40, 41]. Briefly, mice were killed by cervical dislocation under halothane anesthesia, and vibratome-sectioned corticostriatal coronal slices (200 μm) were prepared from fresh tissue blocks of the brain [9, 40, 41]. A single slice was then transferred to a recording chamber and submerged in a continuously flowing artificial CSF (ACSF) (34 °C, 2–3 ml/min) gassed with 95 % O_2_–5 % CO_2_. Whole-cell patch clamp recordings were made in voltage clamp mode, at the holding potential of −80 mV to study spontaneous glutamate-mediated excitatory post-synaptic currents (sEPSCs). Bicuculline (10 µM) (Sigma-Aldrich) was added to the bathing ACSF to block GABA_A_-mediated transmission. Synaptic events were stored using P-CLAMP 9 (Axon Instruments) and analyzed offline on a personal computer with Mini Analysis 5.1 (Synaptosoft) software. The detection threshold of sEPSCs was set at twice the baseline noise. Offline analysis was performed on sEPSCs recorded during fixed time epochs (2–3 min, 3–6 samplings), sampled every 2 or 5 min. Only cells that exhibited stable frequencies (less than 20 % changes during the control samplings) were taken into account. For kinetic analysis, events with peak amplitude between 10 and 50 pA were grouped, aligned by half-rise time, normalized by peak amplitude. For each cell, the events were averaged to obtain rise times, decay times, and half widths. The effects of microglia on striatal excitatory transmission were assessed as described [9] by placing N9 cells (2.5 × 10^5^) onto the surface of a striatal slice, submerged in an oxygenated recording chamber for 30 min before electrophysiological recordings. One to six cells per mouse were recorded. For each group, at least four animals were assessed.

### Statistical analysis

Results are presented as mean ± standard error of the mean (SEM). The difference in means between two groups was assessed by two-tailed Student *t* test. Statistical analysis was performed using Prism 5 (GraphPad Software). *P* values were considered significant at *P* < 0.05 and highly significant at *P* < 0.01 and *P* < 0.001.

For the electrophysiological studies, statistical analysis was performed using paired or unpaired Student *t* test if comparisons were between two groups. Multiple comparisons were analyzed by one-way ANOVA for independent measures followed by Tukey’s honest significance test.

## Results

### Upon in vitro exposure to MMF, LPS-activated microglia downregulate expression of inflammatory molecules and upregulate expression of alternative activation markers

As a pre-requisite to in vitro investigation of MMF effect on microglia, we evaluated the potential cytotoxicity of MMF, the bioactive metabolite of DMF, on N9 cells by measuring viability and proliferation upon 24-h exposure to increasing concentrations of MMF. MTT assay results show that at the selected range (1–100 μM) MMF was not toxic for N9 cells (Supplementary Fig. S1a). To select the optimal effective concentration of MMF, we analyzed the mRNA expression by MMF-treated and untreated LPS-activated N9 cells of genes representative of classical (tumor necrosis factor α, Tnf; nitric oxide synthase inducible, Nos2) and alternative activation (chemokine (C-X3-C motif) receptor 1, Cx3cr1; nuclear receptor related 1 protein, Nr4a2) phenotypes. We observed that, while Tnf and Nos2 were downregulated at all MMF concentrations tested, the lowest one, 1 μM, was the most effective in reducing the expression of Cx3cr1 and Nr4a2 to basal levels (Supplementary Fig. S1b); the effect of MMF on microglia activation was therefore tested at this concentration in all subsequent in vitro studies.

**Fig. 1:**
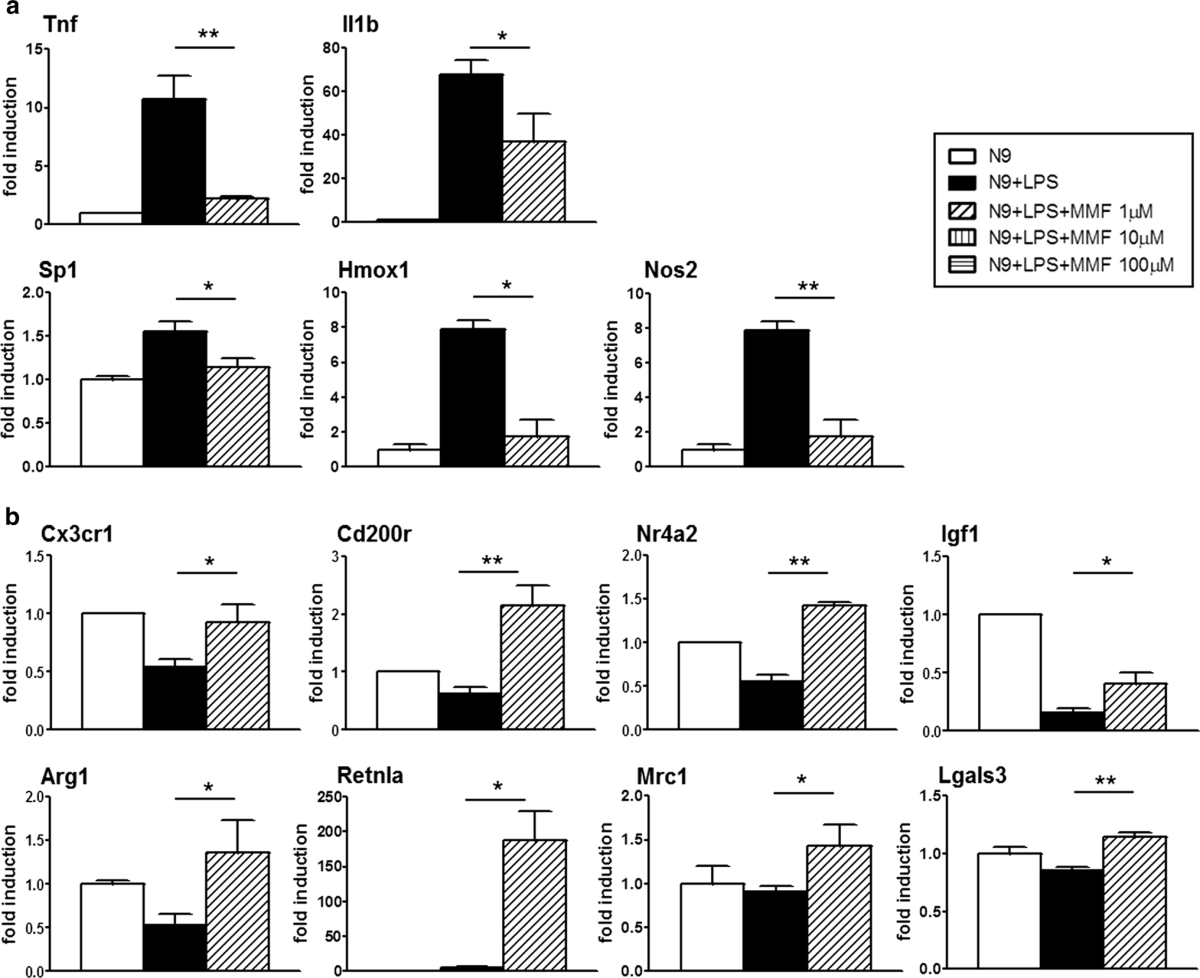
MMF induces a molecular switch in activated microglia from a pro-inflammatory to an alternatively activated phenotype. Real-time PCR analysis of mRNA expression of relevant genes in LPS-activated N9 cells treated with MMF shows that **a** MMF decreases the expression of genes associated with a pro-inflammatory phenotype in activated microglia; and **b** MMF upregulates the expression of neuroprotective (alternatively activated) phenotype markers in activated microglia. Data are presented as fold induction over the expression of the genes in untreated N9 cells, and results are shown as mean ± SEM of at least three independent experiments. *P < 0.05 and **P < 0.01

The effect of MMF on expression of molecules associated with a pro-inflammatory microglial phenotype is shown on Fig. 1a. Exposure to MMF led to a full reversal to basal expression levels or strong downregulation of the genes coding for the inflammatory cytokines, Tnf and interleukin-1β (Il1b), the myeloid transcription factor, 31 kDa-transforming protein/SFFV proviral integration 1 protein (Spi1, also known as PU.1), which is critical for the viability and effector functions of microglia, and the stress response molecules heme oxygenase-1 (Hmox1) and Nos2, all of which were strongly upregulated by N9 cells upon LPS activation (Fig. 1a). In parallel, the expression of chemokine (C-X3-C motif) receptor 1 (Cx3cr1) and CD200 receptor (Cd200r), receptors essential for the maintenance of interactions with ligands expressed by neurons and whose disruption results in highly activated neurotoxic microglia [8, 61], as well as of that of Nr4a2, an orphan nuclear receptor which functions to inhibit expression of pro-inflammatory neurotoxic mediators in both microglia and astrocytes [42], was reversed or increased over basal levels upon exposure of LPS-activated microglia to MMF (Fig. 1b). We subsequently analyzed the effect of MMF on microglial expression of other genes coding for markers associated with an alternative activation phenotype, insulin growth factor 1 (Igf1) [52], arginase 1 (Arg1) [62], and resistin-like alpha found in inflammatory zone (Retnla, also known as Fizz1) [35], and confirmed the effect of MMF in modulating the classically activated LPS-induced microglial phenotype to that of an alternatively activated phenotype, with reversion to basal levels and/or high enhancement of the expression of these factors (Fig. 1b).

These molecular data therefore strongly support our hypothesis that MMF acts on activated microglia by inducing a switch from a classically activated, pro-inflammatory to an alternatively activated, potentially neuroprotective phenotype.

### MMF induces functional modifications commensurate with an alternative activation phenotype in LPS-activated microglia in vitro

**Fig. 2:**
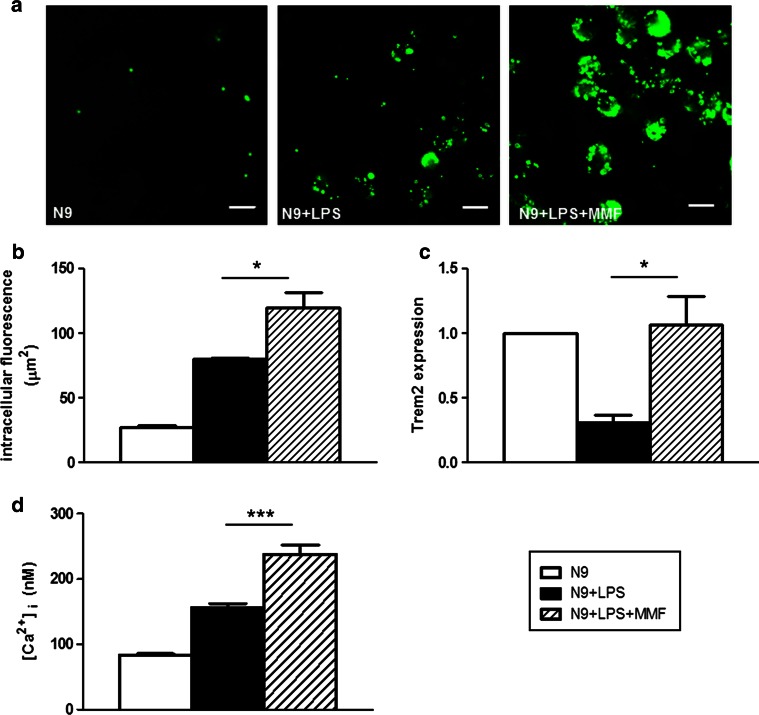
MMF prompts functional changes associated with an alternatively activated phenotype of microglia. MMF enhances the phagocytic activity of LPS-activated N9 cells in vitro as shown by confocal microscopy images of LPS-activated N9 cells treated or not with MMF in the presence of green fluorescent microspheres (**a**) and quantified as the cell area occupied by the microspheres (**b**); the average cell section area was 34 ± 1 and 180 ± 7 μm^2^ for non-activated and activated cells, respectively. **c** MMF upregulates mRNA expression of Trem2 in LPS-activated N9 cells. Data are presented as fold induction over the expression of Trem2 in untreated N9 cells. **d** MMF increases [Ca^2+^]_i_ in LPS-activated N9 cells, as measured by fluorometric analysis of Fura 2AM. Results are shown as mean ± SEM of at least three independent experiments. *P < 0.05 and ***P < 0.001. *Bar* = 15 μm

To evaluate if the molecular changes observed in activated microglia upon exposure to MMF correlates with relevant functional modifications, we analyzed phagocytic function and intracellular Ca^2+^ concentration ([Ca^2+^]_i_), which are linked to executive microglia function such as release of pro- and anti-inflammatory cytokines, nitric oxide or trophic factors [7]. In LPS-activated N9 cells, exposure to MMF further enhanced the phagocytosis in vitro of fluorescent microbeads (Fig. 2a, b). Most importantly, this increased phagocytic activity was associated with an increase in the expression of triggering receptor expressed by myeloid cells-2 (Trem2) mRNA, which codes for a molecule involved in the clearance of apoptotic neurons and myelin debris by microglia in the absence of inflammation [21, 50], and which was downregulated upon microglial activation (Fig. 2c).

Fluorometric determination of [Ca^2+^]_i_ in N9 cells incubated with the fluorescent calcium indicator Fura 2 AM demonstrated an increased [Ca^2+^]_i_ upon activation with LPS, and this increase was further enhanced when the activated cells were treated with MMF (Fig. 2d).

These data indicate that the molecular phenotypic changes induced by MMF are accompanied by functional changes that are consistent with the phenotype of microglia involved in the maintenance of CNS homeostasis such as phagocytosis in the absence of inflammatory responses.

### The anti-inflammatory action of MMF is mediated through signaling via HCAR2

**Fig. 3:**
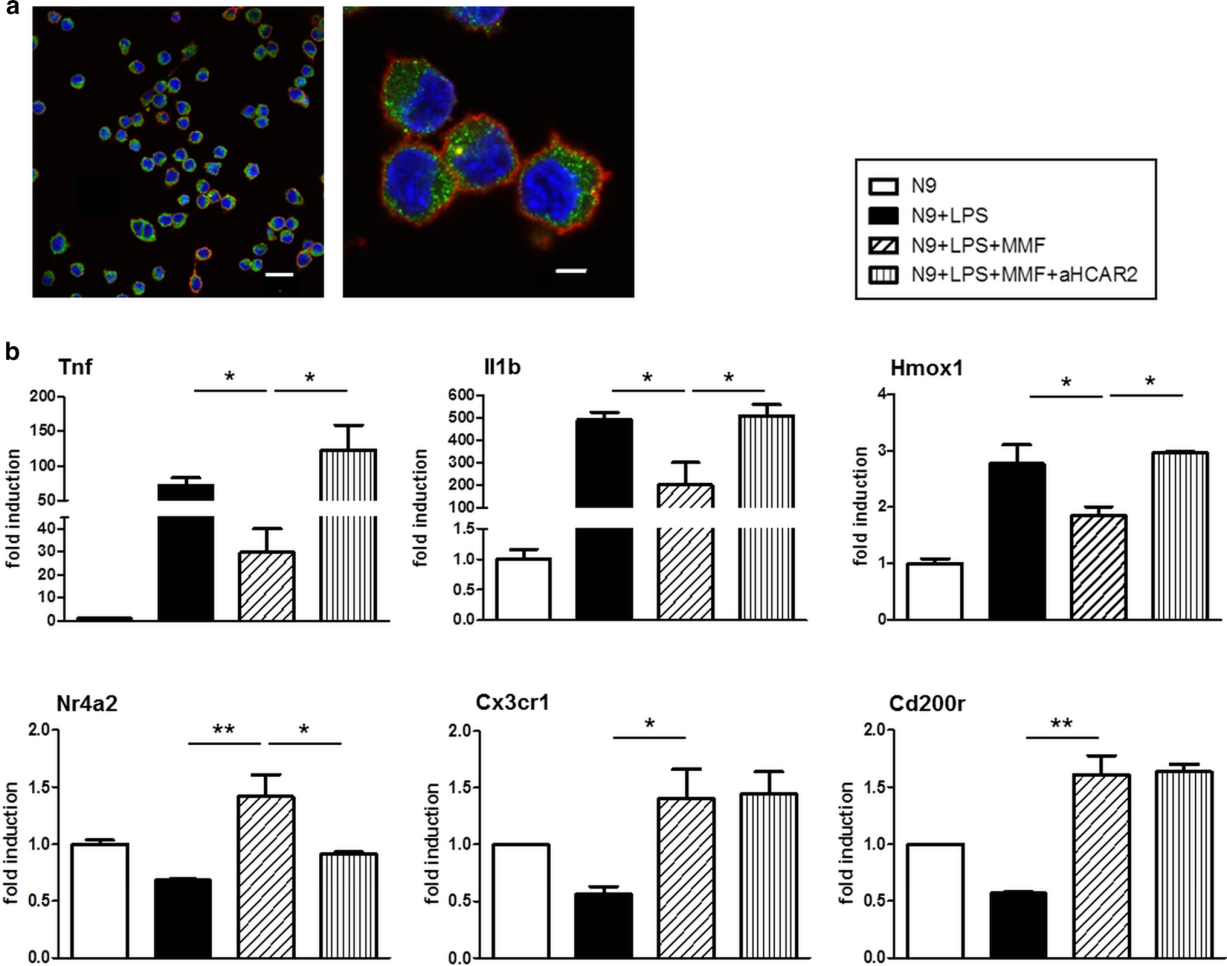
MMF signals through binding to HCAR2. **a** N9 cells express HCAR2. Confocal microscopy images of N9 cells stained with anti-HCAR2 (*green*) and anti-CD11b (*red*) antibodies, and with the nuclear marker DAPI (*blue*). *Bars* = 20 and 5 μm for *left* and *right* *panels*, respectively. Magnification = 40× and 100× for *left* and *right panels*, respectively. **b** Blocking HCAR2 reverts the effect of MMF on the expression of genes dependent on NF-κB, but not on that of Cx3cr1 and Cd200r. N9 cells were incubated with or without anti-HCAR2 antibody (aHCAR2) for 1 h prior to LPS activation for 6 h in the presence or absence of MMF, and mRNA expression of the indicated genes was assessed by real-time PCR. Data are presented as fold induction over the expression of the genes in untreated N9 cells and results are shown as mean ± SEM of at least three independent experiments. *P < 0.05, **P < 0.01

While the mechanisms of action of DMF and of its metabolite MMF are only partially understood, MMF was recently shown to be a potent agonist of the hydroxycarboxylic acid receptor, HCAR2 [20, 51]. We therefore hypothesized that the activity of MMF on microglia may involve binding to, and activation of, this receptor. While HCAR2 was shown to be expressed by a large number of cells, including immune cells [12, 20, 29, 58], its expression in microglia has not been directly demonstrated at the protein level. We therefore ascertained its expression in murine microglia-derived N9 cells by confocal microscopy analysis upon co-staining with anti-HCAR2 (aHCAR2) and anti-CD11b antibodies. As can be seen in Fig. 3a, HCAR2 is expressed by N9 cells.

To determine if the modulating effect of MMF on microglia is mediated through binding to HCAR2, we blocked the MMF/HCAR2 interaction using aHCAR2, as specific antagonists for HCAR2 are not available, and tested the expression of genes coding for molecules associated with the classical activation phenotype, Tnf, Il1b, and Hmox1, as well as molecules associated with the alternative activation phenotype, Nr4a2, Cx3cr1, and Cd200r. Antibody blockade of HCAR2 fully reversed the effect of MMF on the mRNA expression of inflammatory and oxidative response molecules, Tnf, Il1b, and Hmox1, as well as that of Nr4a2 in activated microglia (Fig. 3b). In contrast, exposure to aHCAR2 had no effect on the upregulation of Cx3cr1 and Cd200r expression in MMF-treated activated microglia (Fig. 3b).

We noted that the expression of many of the tested genes modulated by MMF exposure were dependent on NF-κB activation, including Tnf, Il1b, Sp1, Hmox1, Nos2, and Nr4a2 [6, 25, 26, 31, 33, 37, 44, 60]. Of these gene transcripts, four that were also tested for their expression in LPS-activated MMF-treated microglia upon blockade of HCAR2, Tnf, Il1b, Hmox1, and Nr4a2 showed reversed expression levels under this condition, whereas the expression of non-NF-κB-dependent Cx3cr1 and Cd200r was unaltered (Fig. 3b); suggesting that the NF-κB pathway is implicated in the modulation of the inflammatory phenotype of microglia by MMF activation of HCAR2 signaling.

### MMF modulates microglia activation phenotype through inhibition of the NF-κB pathway via the AMPK/Sirt1 axis

**Fig. 4:**
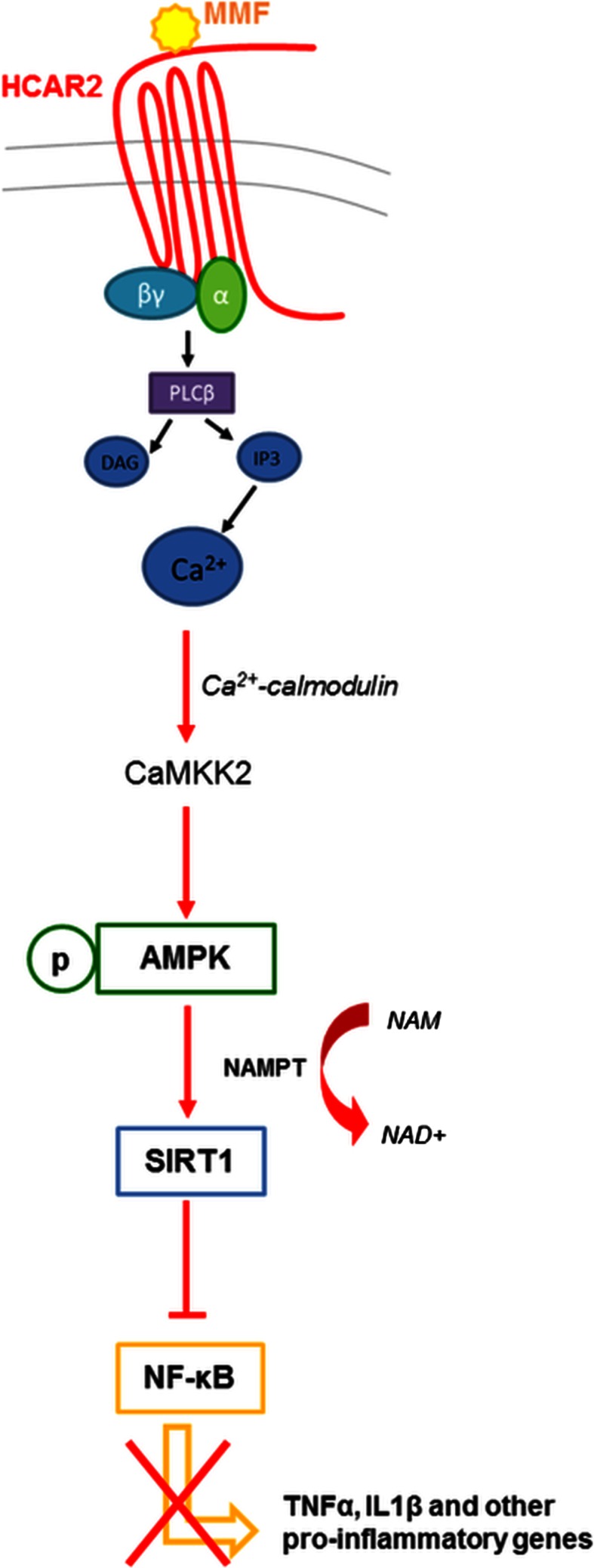
Postulated signaling pathway for MMF through binding to HCAR2

Based on the above demonstration that MMF induced an increase in [Ca^2+^]_i_ and resulted in the inhibition of NF-κB-dependent gene expression, together with previous studies showing that activation of HCAR2 resulted in increases in [Ca^2+^]_i_ [3, 20], we hypothesized that MMF was initiating an HCAR2-dependent downstream signaling pathway activated by the increase in intracellular Ca^2+^ levels (Fig. 4). Thus, activation of HCAR2 upon MMF binding would lead to activation of the G_i_-type G protein signaling cascade; this, in turn, would result in phospholipase C activation, possibly through release of the βγ subunit [4] and, thereby, in increased [Ca^2+^]_i_. The increase in [Ca^2+^]_i_ would lead to activation of AMPK through phosphorylation of threonine 172 by calcium/calmodulin-dependent protein kinase 2 (CaMKK2) [22] whose activity is enhanced by [Ca^2+^]_i_ [1]. It has been shown that the NAD**+**-dependent protein deacetylase sirtuin-1 (SIRT 1) suppresses NF-κB by direct deacetylation at lysine 310 of NF-κB p65 [57]. As its deacetylase activity is regulated by NAD+ availability [38]; we postulated that AMPK activation would lead to an increase in NAD+ generated through phospho-AMPK (p-AMPK)-mediated induction of nicotinamide phosphoribosyltransferase (NAMPT), the rate-limiting enzyme in the NAD biosynthesis pathway [14], and thereby in activation of SIRT1 resulting in inhibition of NF-κB signaling [23, 32].

**Fig. 5:**
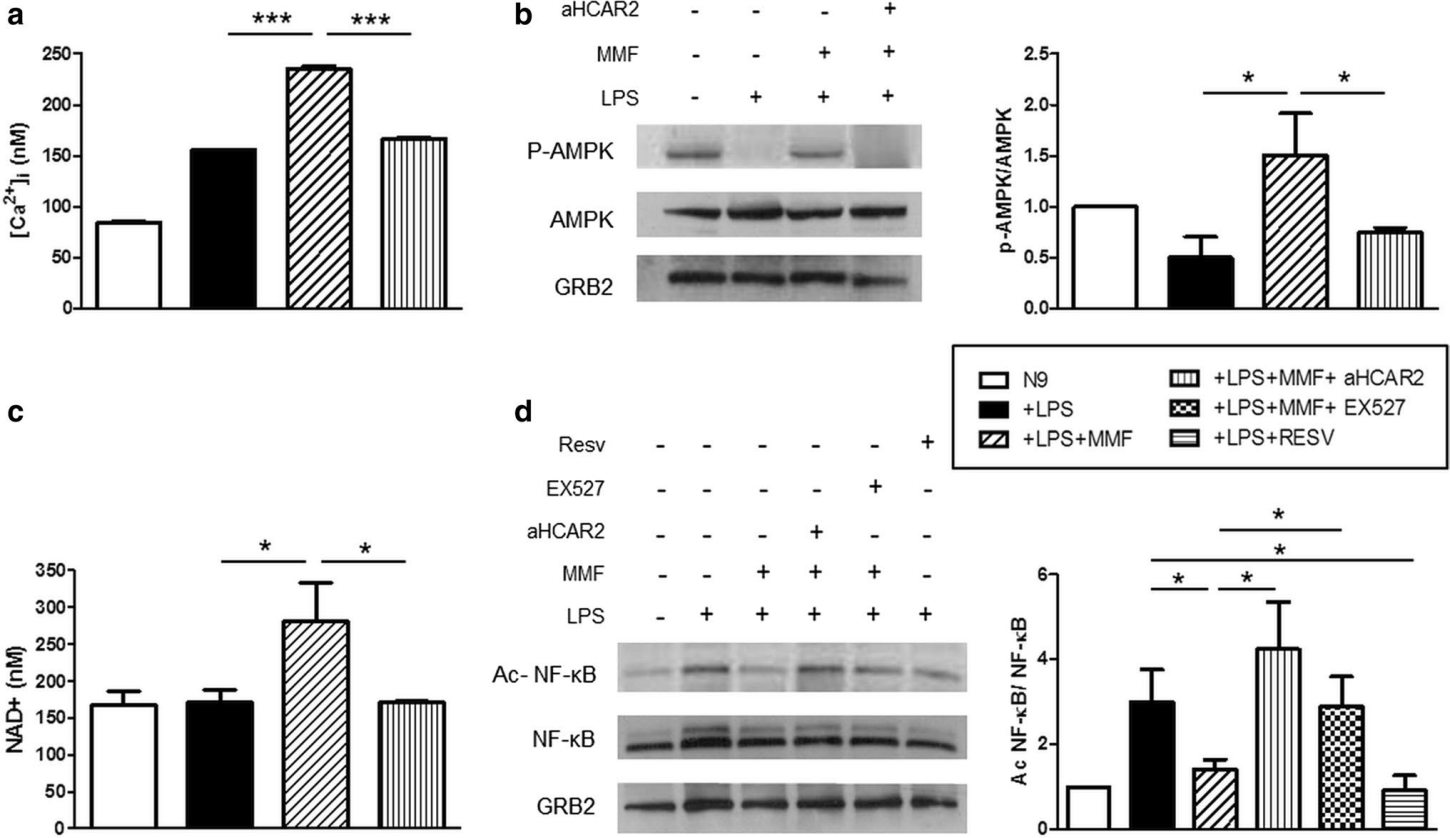
Validation of the HCAR2-mediated pathway for MMF signaling. **a** Blocking HCAR2 suppresses MMF-induced increase in [Ca^2+^]_i_ in activated microglia. N9 cells were incubated with or without aHCAR2 for 1 h prior to LPS activation for 6 h in the presence or absence of MMF and [Ca^2+^]_i_ was determined by fluorimetric analysis. **b** MMF treatment activates AMPK in LPS-activated microglia. Representative Western blot (*left panel*) and quantification (*right panel*) of at least three Western blot analyses of N9 cell lysates are shown. N9 cells were incubated or not with aHCAR2 prior to activation with LPS for 30 min in the presence or absence of MMF. Western blot analysis of the lysates was performed with anti-p-AMPK, which recognizes phosphorylated Thr172, anti-AMPK, and anti-GRB2 (as loading control) antibodies. Quantification by densitometric analysis of Western blot films is presented as the proportion of p-AMPK over total AMPK (fold induction over N9), normalized to GRB2. Results are shown as mean ± SEM of at least three independent experiments; *P < 0.05. **c** MMF treatment increases NAD^+^ levels in LPS-activated microglia. *Graph* represents colorimetric quantification of intracellular NAD^+^ produced by N9 cells incubated with or without aHCAR2 prior to activation with LPS in the presence or absence of MMF for 30 min. **d** MMF treatment leads to NF-κB inhibition in activated microglia through deacetylation of NF-κB p65. Representative Western blot (*left panel*) and quantification (*right panel*) of at least three Western blot analyses of N9 cell lysates are shown. N9 cells were incubated with or without aHCAR2 and/or EX527 (10 μM) for 1 h prior to activation with LPS in the presence or absence of MMF or Resv (50 μM) for 6 h. Western blot analysis was performed with anti-Ac-NF-κB antibody, which recognizes acetylated lysine 310 of NF-κB p65, anti-NF-κB, and anti-GRB2 (as loading control) antibodies. Quantification by densitometric analysis of Western blot films is presented as the proportion of Ac-NF-κB over total NF-κB (fold induction over N9). Results are shown as mean ± SEM of at least three independent experiments; *P < 0.05

As the first step towards validating this possible pathway, we demonstrated that the increased [Ca^2+^]_i_ is indeed a result of MMF binding to HCAR2, as blocking the receptor with aHCAR2 prevented the MMF-induced [Ca^2+^]_i_ flux observed in Fura 2AM-treated LPS-activated N9 cells upon exposure to MMF (Fig. 5a).

We then quantified p-AMPK by Western blotting to assess AMPK activation as the first downstream component of the pathway and observed a decrease in the proportion of p-AMPK in LPS-activated N9 cells. In the presence of MMF, however, there was a clear increase in p-AMPK, and this increase was inhibited upon blocking HCAR2 (Fig. 5b).

The ensuing step of this pathway implicates an activation of NAMPT that converts NAM to NAD+, essential for the activation of SIRT1, and we measured NAD+ concentration in LPS-activated N9 cells exposed, or not, to MMF and upon antibody blockade of HCAR2. As can be seen in Fig. 5c, the expected increase in NAD+ upon exposure to MMF and its reversal in the presence of aHCAR2 support an activation of NAMPT in the HCAR2 pathway triggered by MMF.

To validate the last step of the pathway, which implicates the activation of the NAD+-dependent SIRT1 leading to deacetylation of NF-κB and therefore its inhibition, we measured the outcome of the enzymatic reaction, that is we monitored the acetylation status of NF-κB in N9 cells, upon various treatments aimed at assessing the effect not only of blocking MMF binding to HCAR2 with aHCAR2, but also of inhibiting SIRT1 itself with the SIRT1-specific inhibitor, EX527, or activating it directly with a specific activator, Resveratrol (Resv) (Fig. 5d). Densitometric quantification of Western blots demonstrated that the increase in Ac-NF-κB upon LPS activation was reversed by MMF and that this effect was abrogated in presence of aHCAR2, suggesting that SIRT1 activation is implicated in MMF effect via HCAR2 signaling. Along those lines, exposure of LPS-activated MMF-treated N9 cells to an inhibitor of SIRT1, EX527, had a similar effect as HCAR2 blockade (Fig. 5d). As a control to our study and to support the implication of SIRT1 in the effect of MMF, we exposed LPS-activated N9 cells to Resv and observed a reduction in Ac-NF-κB similar to that seen upon exposure to MMF (Fig. 5d).

Altogether, these data support the model in which the anti-inflammatory effects of MMF on microglia are mediated through activation of the AMPK/SIRT1 axis, resulting in inhibition of NF-κB, which controls the expression of multiple inflammatory cytokines and mediators.

### DMF treatment leads to an increased expression of markers of alternatively activated microglia in CNS of EAE mice

**Fig. 6:**
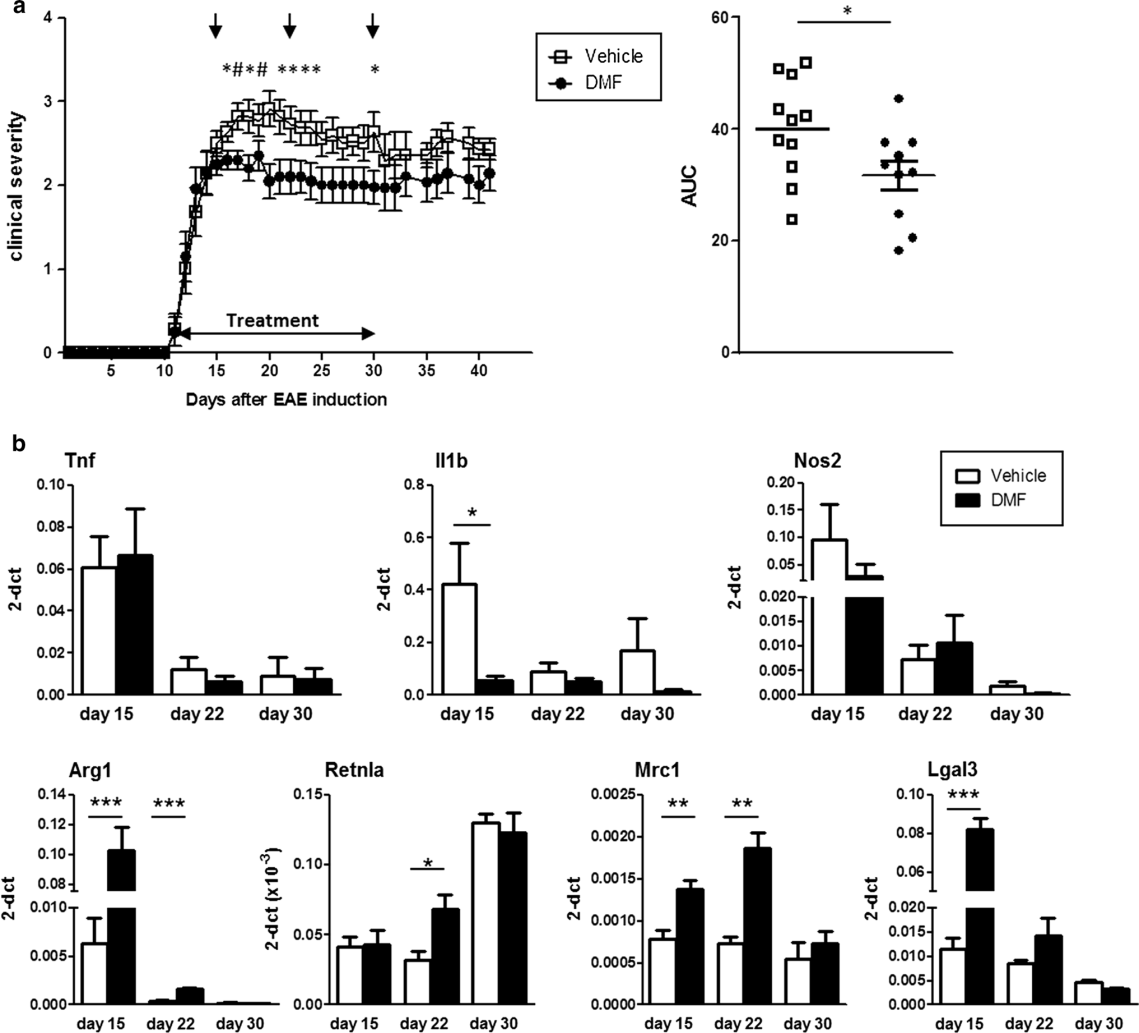
Treatment with DMF ameliorates EAE and induces an increase in markers of alternatively activated microglia in vivo. **a** Administration of DMF at clinical onset reduces EAE severity at post-peak/early chronic phase. C57BL/6J mice (*n* = 20 per group, randomly allocated) were immunized with MOG 35–55 and treated daily by oral gavage with DMF (DMF) or with hydroxypropyl methylcellulose  alone (Vehicle) from the day of disease onset until day 30 post-immunization. Mice (*n* = 3 per group at each time point) were sampled at indicated time points (*arrows*) for PCR analysis of brain mRNA (see below). Data are presented as the mean ± SEM daily clinical score (*left panel*) and as the area under the curve (AUC) of EAE clinical course calculated for each mouse between days 15–30 post-immunization *(right panel*). *P < 0.05, ^#^ *P* < 0.01. **b** In vivo treatment with DMF downregulates the expression of pro-inflammatory genes and upregulates genes of the alternatively activated microglia phenotype in the CNS of EAE mice. RNA was isolated from brains of EAE mice treated or not with DMF at indicated time points corresponding to acute (day 15), post-peak (day 22) and chronic (day 30) phases. Expression of the indicated genes was quantified by real-time PCR. Results are shown as mean ± SEM. *P < 0.05, **P < 0.01

To ascertain whether or not the effects of MMF that we had observed in vitro are recapitulated in vivo, we tested the therapeutic effect of its precursor, DMF, in C57Bl/6J mice with chronic EAE induced with MOG peptide. Mice were treated by daily oral gavage with 150 mg/kg body weight DMF suspended in hydroxypropyl methylcellulose  (mean MMF plasma concentration ± SEM: 49.81 ± 9.57 μg/ml at 30 min after dosing; data not shown) from the day following the onset of clinical symptoms until day 30 after immunization when the chronic disease phase is well established. As seen in Fig. 6a, we confirmed the reported beneficial effect of DMF on MOG-induced EAE [28]. Thus, a significantly lower clinical severity was observed in EAE-affected mice treated with DMF from day 16, that is 4 days after the start of treatment, which remained significant until day 22, i.e., throughout the peak/early chronic phase (Fig. 6a, left panel); statistical analysis of the area under the curve (AUC) from days 15 to 30 post-immunization showed significant difference between DMF-treated and untreated mice (Fig. 6a, right panel; *P* < 0.05).

To determine the effect of DMF on the brain environment in EAE mice, we analyzed the mRNA expression of markers for inflammatory molecules, Tnf, Il1b, and Nos2, as well as for markers of alternative activation, Arg1, Retnla, mannose receptor C type 1 (Mrc1; also known as Cd206) and lectin galactose binding, soluble 3 (Lgals3; also known as Gal3) in brain from three DMF-treated (DMF) mice and three untreated (Vehicle) mice (Fig. 6b), at relevant time points corresponding to acute (day 15), post-peak/early chronic (day 22), and chronic (day 30) phases. While there was no significant difference in brain Tnf expression between treated and untreated mice at any time point tested, the expression of Il1b was significantly downregulated in DMF-treated mice at day 15; similarly, albeit not significant, there was a trend towards lower expression of Nos2 at the same time point. Markers of alternatively activated phenotype were significantly increased in brain of DMF-treated EAE-affected mice in the early phase of disease (Fig. 6b). By day 30, at the established chronic phase when a significant difference in disease severity was no longer detected between DMF-treated and untreated mice, the expression levels of all these markers in DMF-treated mice had returned to those of untreated mice (Fig. 6b).

These results confirm that oral administration of DMF to mice with EAE results in clinical amelioration and can induce an increase in the expression of markers of an alternatively activated phenotype, as observed in microglia exposed in vitro to MMF.

### Validation of DMF effect at the synaptic level: DMF protects glutamatergic synapses

**Fig. 7:**
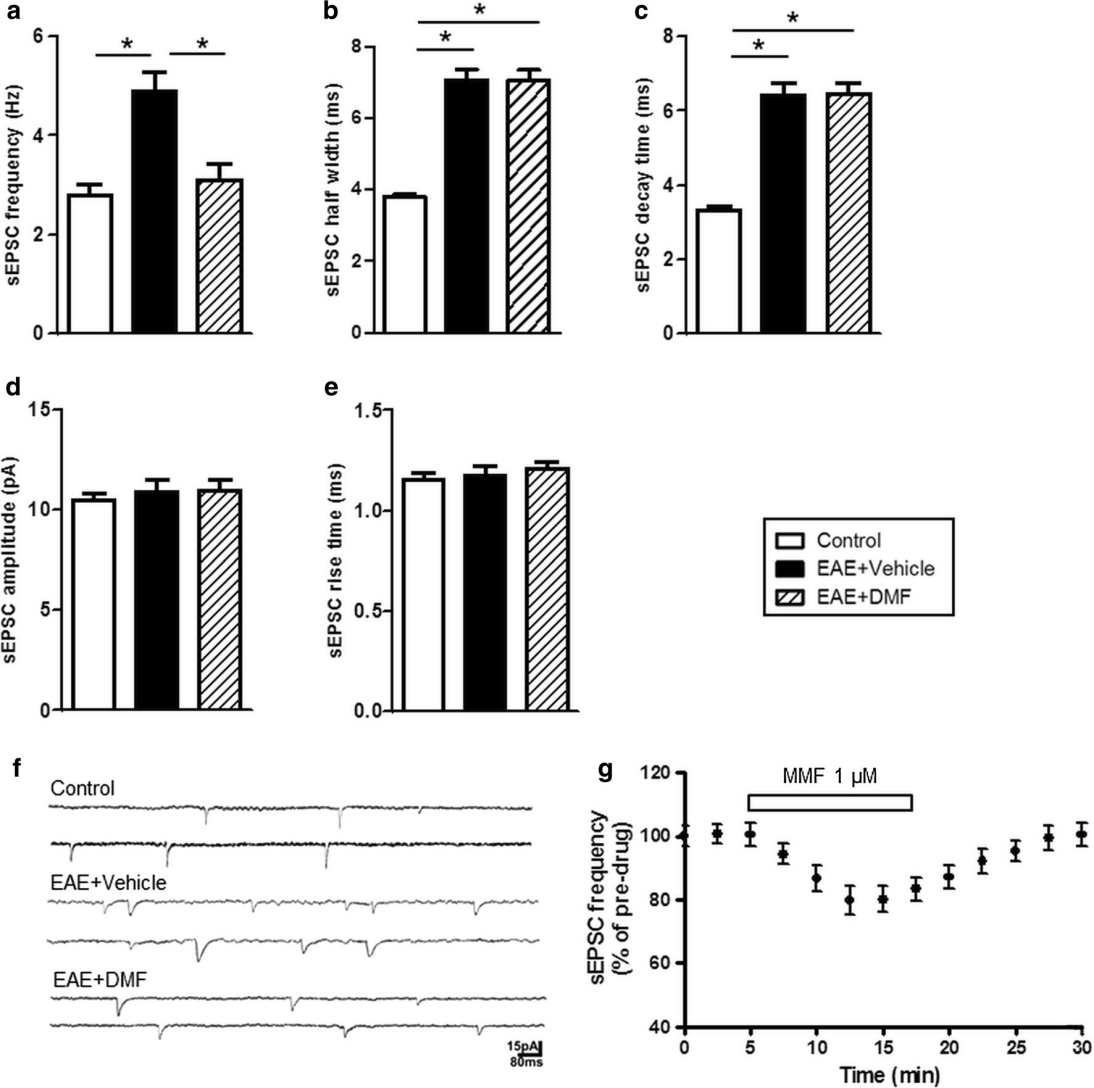
DMF treatment normalizes pre-synaptic abnormalities of glutamatergic transmission in EAE mice. **a**–**e** DMF treatment normalizes the frequency, but not the increased decay time and half-width of glutamatergic sEPSCs of EAE mice. sEPSC recordings from single striatal neurons (*n* = 12 neurons for a total of four mice for each group) were performed on EAE mice sampled between day 20 and day 25 post-immunization. Corticostriatal slices obtained from naïve mouse brain (Control) were used for control recordings (*n* = 14 neurons recorded for a total of four mice). *P < 0.05. **f** Representative electrophysiological traces of voltage clamp recordings. **g** MMF applied directly on control corticostriatal slices induces significant reduction of sEPSC frequency. MMF (1 μM) was added to the bathing ACSF from the indicated time and continuously for 12 min. Recordings were performed on single striatal neurons (*n* = 10)

In EAE, neuroinflammation enhances glutamate transmission and promotes synaptopathy, which occurs in the early phase of disease and is associated with the release of inflammatory cytokines, such as TNFα and IL-1β, from activated microglia [9, 30]. To understand whether or not DMF might affect EAE-mediated synaptopathy, we have performed whole-cell patch clamp electrophysiological recordings from single neurons in corticostriatal slices from EAE mice, treated or not with DMF, and control mice, between 20 and 25 days post-immunization (post-peak/early chronic phase of disease). As in our previous studies [9, 40, 41], we observed pre- and post-synaptic abnormalities of glutamatergic transmission in EAE mice (Fig. 7). Thus, both frequency and kinetic properties of glutamate-mediated sEPSCs were altered in EAE mice, as compared to that in control mice, with a longer decay time accounting for increased sEPSC duration. DMF treatment normalized sEPSC frequencies (*P* < 0.05 as compared to vehicle-gavaged EAE-affected mice; Fig. 7a), but had no effect on the increase in sEPSC decay time and half-width (Fig. 7b, c, f). Neither rise time nor amplitude of sEPSCs was altered by EAE induction, as previously shown [9], or by DMF treatment (Fig. 7d, e).

These data might indicate that DMF directly alters sEPSCs in EAE mice by modulating basal glutamatergic transmission at central synapses. Thus, we tested the effect of MMF, the active metabolite of DMF, on spontaneous synaptic transmission. Upon exposure of corticostriatal slices from control mice to MMF, we observed a direct effect on neuronal synaptic activity. Thus, MMF at the low concentration (1 μM) significantly reduced sEPSC frequency (*P* < 0.01), but not amplitude (100.8 ± 1.6 vs. 100 % pre-treatment values; data not shown) in all the tested control neurons (Fig. 7g), indicating that MMF directly modulates glutamatergic transmission at the pre-synaptic level.

**Fig. 8:**
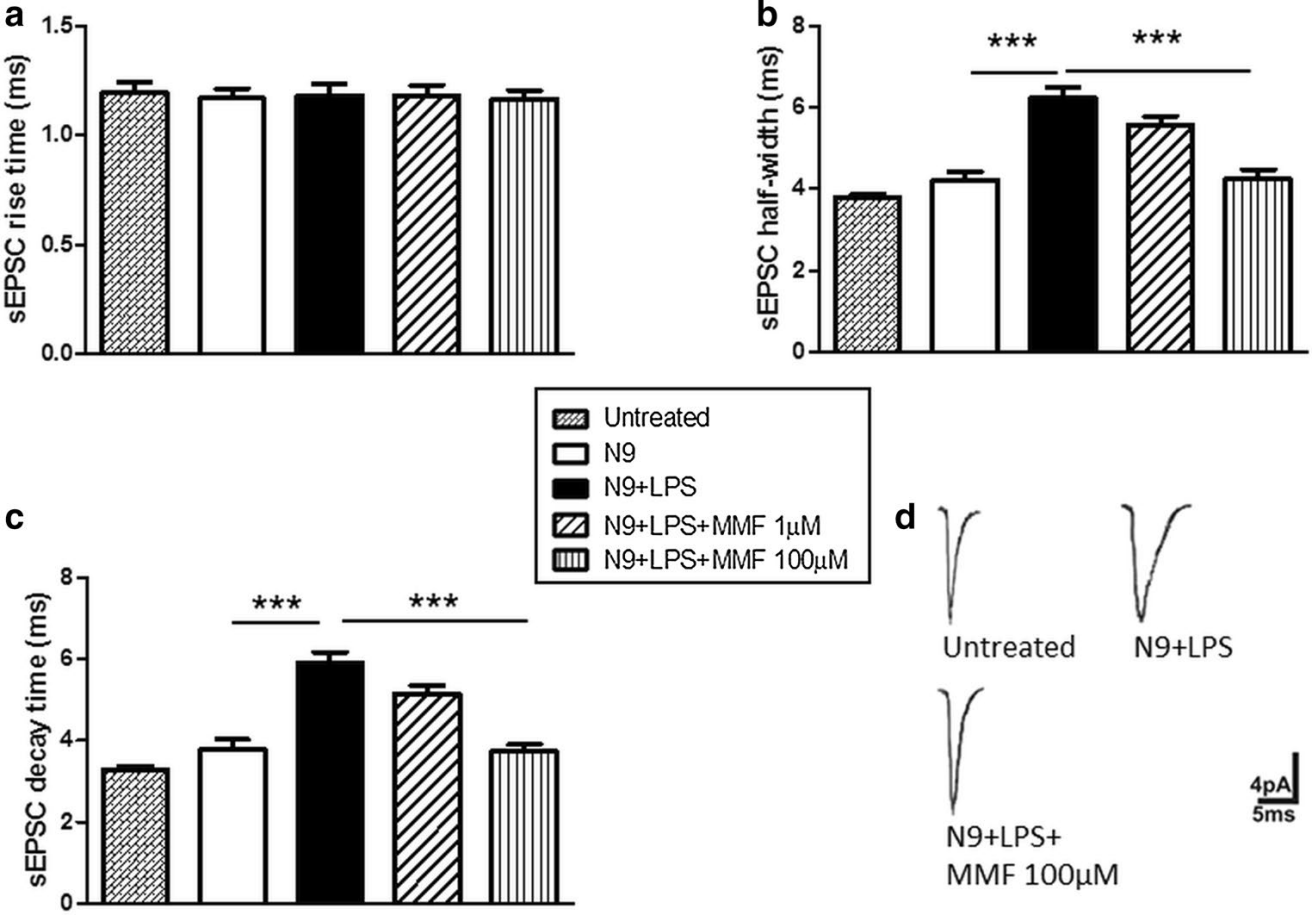
DMF modulates microglia action at synapses. (**a**–**c**) The increase in sEPSC decay time and half-width induced upon exposure of control corticostriatal slices to LPS-activated microglia is reverted when activated microglia are treated with MMF at high concentration. Corticostriatal slices obtained from naïve mouse brain were exposed to microglia activated with LPS in the presence or absence of 1 or 100 μM MMF (N9 + LPS + MMF 1 μM or 100 μM) for 30 min prior to electrophysiological recordings from single striatal neurons (*n* = 11). Control recordings were obtained from untreated slices (Untreated) or slices exposed to N9 cells (N9) or N9 cells activated with LPS (N9 + LPS); ***P < 0.001. **d** Electrophysiological traces are example of mean peak obtained by group analysis in the presence of activated microglia untreated or treated with high-dose MMF

Alternatively, DMF might also alter sEPSCs in EAE mice by an indirect immunomodulatory mechanism through its effect on microglia. As the exposure of striatal slices from control mice to activated microglia results in altered glutamate transmission such as seen in EAE, an effect that can be attributed to TNFα released from the activated microglia [9], we directly tested the effects of MMF on microglia action at synapses. While non-activated N9 cells had no effect on the physiological properties of striatal sEPSCs recorded from control slices (Fig. 8a–c), exposure of control slices to LPS-activated N9 cells significantly increased the duration of sEPSCs (*P* < 0.001; Fig. 8b, d), by slowing their decay phases (*P* < 0.001; Fig. 8c, d). We hypothesized that the lack of protective effects of DMF on sEPSC duration in EAE mice could be related to a low central exposure of MMF after oral gavage (dose-limiting toxicity prevents testing higher chronic doses). Indeed, treatment of activated microglia with a high concentration of MMF (100 μM) fully prevented the increase of sEPSC duration, whereas their treatment with a low concentration (1 μM) had no such effect (Fig. 8b–d).

These findings support the concept that the neuroprotective effect of DMF is exerted on neurons directly at pre-synaptic terminals by modulating glutamate release, and indirectly at post-synaptic level, by modulating microglia function in a dose-dependent manner.

## Discussion

In this study, we show that the therapeutic effect of DMF, which leads to neuroprotection in EAE, could be mediated at the level of the CNS not only by directly affecting neurons through induction of the Nrf2-mediated anti-oxidative pathway [28], but also through direct modulation of the activation state of microglia, inducing reversal, or a strong downregulation, of activation-induced expression of inflammatory and stress response genes and upregulation of genes associated with an alternatively activated and therefore neuroprotective phenotype of microglia. Moreover, we postulate and validate an Nrf2-independent pathway triggered by MMF binding to HCAR2 through which DMF can act to switch microglia phenotype from pro- to anti-inflammatory, thereby indirectly affecting neuronal survival and function.

The anti-inflammatory action of DMF/MMF on microglial cells is known, with in vitro studies on LPS-activated mixed glial cells, astrocytes and microglia, showing reversal of the LPS-induced release of inflammatory cytokines [54, 55], but it has been generally attributed to the anti-oxidative action of DMF through activation of the Nrf2 pathway, as occurs in neurons [28]. However, studies have shown that DMF also inhibits NF-κB [27, 47], one of the most potent activating transcription factors of pro-inflammatory genes. While Nrf2 can modulate NF-κB activity within a regulatory feedback loop [11], the evidence for the possible implication of Nrf2 activation in MMF effect on microglia is scant and poor [28, 54, 55]. We hypothesized that Nrf2-independent pathways, which were recently demonstrated in Nrf2^−/−^ mice [46], might also be implicated in immune modulation by DMF. We therefore examined the possibility that a pathway resulting in inhibition of NF-κB, and thereby of relevant inflammatory molecules, could be triggered by MMF binding to HCAR2. Indeed, HCAR2 is implicated in the flushing reaction to DMF [20], and MMF has been identified as a potent HCAR2 agonist [51]. Our immunohistochemical demonstration of the presence of HCAR2 on microglia corroborated the recent report that in the ischemic mouse brain, HCAR2 is expressed exclusively by CD11b+ microglia but not by astrocytes or neurons [36]. We used the anti-HCAR2 antibody to block our postulated pathway whereby activation of HCAR2 by MMF leads to deacetylation, and thereby inhibition, of NF-κB via the AMPK/SIRT1 axis triggered by the increase in intracellular calcium known as a consequence of HCAR2 signaling [51]. At each relevant step analyzed, blockade of HCAR2 reversed the effect of MMF on the relevant pathway component, demonstrating that MMF does signal through HCAR2 to modulate the expression of molecules dependent upon NF-κB activation. In this context, it is relevant to note that the expression of Hmox1, which is upregulated upon activation of Nrf2 [24] but whose expression is also dependent upon NF-κB [25], was downregulated upon triggering of the HCAR2 pathway by MMF in microglia. The possibility that MMF can modulate microglia activation through binding to HCAR2 is of utmost relevance in the context of accumulating evidence implicating HCAR2 signaling in the regulation of immune cell function and inflammation in general. HCAR2 is expressed in various immune cells [5] and studies with the HCAR2 ligand, nicotinic acid, have demonstrated the anti-inflammatory effect of HCAR2 activation. In particular, Zandi-Nejad et al. [58] showed that activated macrophages are subject to negative feedback mechanism via HCAR2 signaling, with suppression of LPS-induced pro-inflammatory cytokine production upon exposure to nicotinic acid, an effect that correlated with NF-κB inactivation. Interestingly in the context of our present study, HCAR2 activation in bone marrow-derived inflammatory macrophages that infiltrate the brain in a mouse model of stroke induces a neuroprotective phenotype in these cells [36]. Similar anti-inflammatory effects associated with activation of HCAR2 in macrophages were demonstrated in a model of atherosclerosis in chimeric mice reconstituted with wild-type vs. HCAR2-deficient bone marrow, where the strong lipid-independent inhibitory effect of nicotinic acid on the progression of atherosclerosis was shown to be mediated by its receptor HCAR2 on bone marrow-derived cells [29]. It is interesting to note in this context that activation of the AMPK/SIRT1 axis antagonizes fatty acid-induced inflammation in macrophages [56]. Importantly, recent studies have also suggested a potential anti-inflammatory role for the endogenous ligand for HCAR2, butyrate [5, 49]. Thus, it is noteworthy that butyrate induces the differentiation of regulatory T cells in vitro and in vivo [2, 15], and ameliorated the development of colitis induced by adoptive transfer of CD4(+) CD45RB(hi) T cells in Rag1(−/−) mice [15]. A recent study showed that butyrate, produced in the colon by gut microbiota, induced the expression of anti-inflammatory molecules in colonic macrophages and dendritic cells in an HCAR2-dependent manner enabling them to promote the differentiation of naïve T cells into regulatory T cells and protecting them against colitis [48]. Our data therefore reinforce the importance of the HCAR2 pathway in inflammation and also suggest its potential relevance for neuroinflammation mediated by microglia. Our data also suggest that administration of DMF, which, after hydrolysis to MMF, crosses the blood–brain barrier, is capable of dampening such neuroinflammation, thereby adding to the neuroprotective action recently proposed for DMF [28]. As we were completing the ex vivo experiments of this study, Chen et al. [10] published their report on EAE in Hcar2^−/−^ mice which confirms a role for HCAR2 signaling in the effect of DMF. Using a preventive treatment paradigm, they observed that, while DMF administration in wild-type mice strongly downregulated disease expression as expected, it had no effect on EAE induced in Hcar2^−/−^ mice, either at the clinical or neuropathological level. Through adhesion and migration assays of purified neutrophils from wild-type and Hcar2^−/−^ mice, they attributed the protective effect of DMF in EAE to inhibition of neutrophil adhesion to brain endothelial cells, and thereby their extravasation into brain tissue, through activation of HCAR2, as reported for other G_i_-coupled receptors [10]. While Chen et al. observed HCAR2 expression on macrophages/microglia, they did not address the possibility that DMF might modulate microglia activation through HCAR2 activation.

We confirm the data of Linker et al. [28] that therapeutic administration of MMF in ongoing disease alleviates EAE. Our data further show that such amelioration is associated with the promotion of a less inflammatory environment in the CNS of treated mice and with the upregulation of microglia markers typical of an alternatively activated homeostatic phenotype, an observation commensurate with previous demonstration that DMF treatment of mice induced to develop EAE results in reduced macrophage/microglia infiltration/migration at early disease phase [28, 45].

In the brain parenchyma, microglia interact intimately with neurons, contacting synaptic elements, and evidence is accumulating for a role of microglia in monitoring and eliminating synapses, thereby contributing to neuronal circuitry [7]. Thus, upon transient cerebral ischemia, the duration of microglia–synapse contacts was markedly prolonged and frequently followed by the disappearance of the pre-synaptic bouton [53]. We have shown that inflammation in EAE triggers alteration of pre- and post-synaptic sites of glutamate synapses, an effect that could be attributed to activated microglia, as the effect of exposure of striatal slices from control mice to microglia activated by pro-inflammatory cytokines mimicked the alterations of glutamate transmission seen in EAE [9]. We have analyzed the possibility that the modulating effect of DMF on microglia activation could be reflected on synapse alterations both in vivo and in vitro. Through whole-cell patch clamp electrophysiological recordings from single neurons in corticostriatal slices, we confirmed the increase in excitatory transmission in ongoing EAE and showed that, while DMF treatment did not affect the half-width and decay time of sEPSCs of striatal neurons in DMF-treated EAE mouse brain, their frequency was reverted to that in control mouse brain, indicating that administration of DMF by oral gavage normalizes pre-synaptic alterations in ongoing EAE, an effect that was corroborated in vitro through exposure of control corticostriatal slices to MMF. That DMF modulates microglial activation in such a way as to revert the neurotoxic effect of activated microglia to a neuroprotective one is reflected by our data showing that, while exposure of control corticostriatal slices to LPS-activated microglia led to an increase in sEPSC half-width and decay time, representative of altered glutamatergic transmission such as seen in EAE and reported previously [9], treatment of the activated microglia with MMF reverted such an effect. These data therefore indicate that MMF can act at post-synaptic level to normalize glutamate transmission indirectly through its modulating effect on microglia.

Altogether, the data reported here demonstrate a possible mode of action for DMF in inflammatory neurodegenerative diseases through its direct effect on inflammatory microglia, which is potentiated by its capacity to cross the blood–brain barrier as shown by pharmacological evidence of the presence of MMF in murine brain upon oral gavage at concentrations commensurate with those shown to affect microglia activation in the present study. Most importantly, our data demonstrate HCAR2 signaling, which has been increasingly implicated in inflammatory processes, as the relevant AMPK/SIRT1-mediated pathway for the effect of MMF on inflammatory microglia, suggesting that HCAR2 signaling is not only relevant to regulate immune cells at the periphery, but also in the CNS.

## Electronic supplementary material

Supplementary material 1 (TIFF 10750 kb) Fig. S1 (**a**) MMF does not affect microglia viability. Growth/viability of N9 cells treated with MMF for 24 h at different concentrations (100 μM, 10 μM and 1 μM) was analyzed by MTT cell viability assay. Data of at least three independent experiments are presented as mean ± SEM percent viability measured by optical density (OD) of MMF-treated cells over OD of control cells (100 %). Results are shown as mean ± SEM of at least three independent experiments. (**b**) The optimal effective concentration of MMF is 1 μM. The expression of representative genes of pro-inflammatory (Tnf, Nos2) and alternatively activated (Cx3cr1, Nr4a2) phenotype of microglia was assessed by real-time PCR; N9 cells were activated with 1 μg/ml LPS and treated with the different concentrations of MMF as above for 24 h. Data are presented as mean fold induction. Single fold induction values did not differ by more than 20 %
