# Supplementary figures and images for: Fumarates modulate microglia activation through a novel HCAR2 signaling pathway and rescue synaptic dysregulation in inflamed CNS
Source: Acta Neuropathol. 2015 Apr 29;130(2):279–95. doi: 10.1007/s00401-015-1422-3 (PMC4503882; doi:10.1007/s00401-015-1422-3)

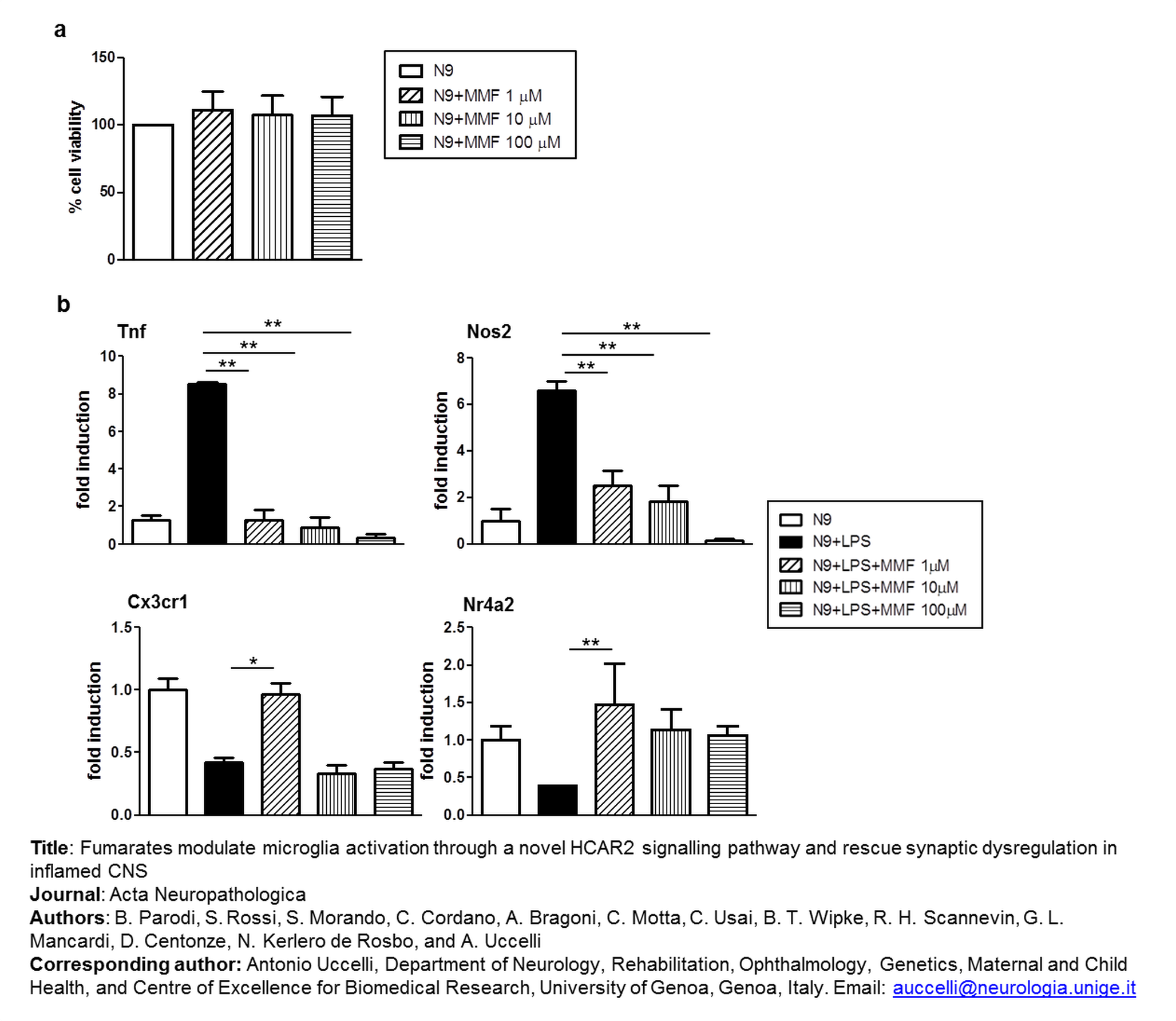

Supplement: Supplementary file 1 — Supplementary material 1 (TIFF 10750 kb) Fig. S1 (a) MMF does not affect microglia viability. Growth/viability of N9 cells treated with MMF for 24 h at different concentrations (100 μM, 10 μM and 1 μM) was analyzed by MTT cell viability assay. Data of at least three independent experiments are presented as mean ± SEM percent viability measured by optical density (OD) of MMF-treated cells over OD of control cells (100 %). Results are shown as mean ± SEM of at least three independent experiments. (b) The optimal effective concentration of MMF is 1 μM. The expression of representative genes of pro-inflammatory (Tnf, Nos2) and alternatively activated (Cx3cr1, Nr4a2) phenotype of microglia was assessed by real-time PCR; N9 cells were activated with 1 μg/ml LPS and treated with the different concentrations of MMF as above for 24 h. Data are presented as mean fold induction. Single fold induction values did not differ by more than 20 % [file 401_2015_1422_MOESM1_ESM.tiff]
